# Androgen receptor expresion in breast cancer: Relationship with clinicopathological characteristics of the tumors, prognosis, and expression of metalloproteases and their inhibitors

**DOI:** 10.1186/1471-2407-8-149

**Published:** 2008-05-28

**Authors:** Luis O Gonzalez, Maria D Corte, Julio Vazquez, Sara Junquera, Rosario Sanchez, Ana C Alvarez, Juan C Rodriguez, Maria L Lamelas, Francisco J Vizoso

**Affiliations:** 1 Unidad de Investigación, Hospital de Jove, Gijón, Spain; 2 Instituto Universitario de Oncología del Principado de Asturias, Oviedo, Spain; 3 Servicio de Anatomía Patológica, Hospital de Jove, Gijón, Spain; 4 Servicio de Ginecología, Hospital Álvarez Buylla, Mieres, Spain; 5 Servicio de Cirugía General, Hospital de Jove, Gijón, Spain; 6 Servicio de Ginecología, Hospital de Jove, Gijón, Spain

## Abstract

**Background:**

In the present study we analyze, in patients with breast cancer, the tumor expression of androgen receptors (AR), its relationship with clinicopathological characteristics and with the expression of several matrix metalloproteases (MMPs) and their inhibitors (TIMPs), as well as with prognosis.

**Methods:**

An immunohistochemical study was performed using tissue microarrays and specific antibodies against AR, MMPs -1, -2, -7, -9, -11, -13, -14, and TIMPs -1, -2 and -3. More than 2,800 determinations on tumor specimens from 111 patients with primary invasive ductal carcinoma of the breast (52 with axillary lymph node metastases and 59 without them) and controls were performed. Staining results were categorized using a score based on the intensity of the staining and a specific software program calculated the percentage of immunostained cells automatically.

**Results:**

A total of 83 cases (74.8%) showed a positive immunostaining for AR, but with a wide variation in the staining score values. There were no significant associations between the total immunostaining scores for AR and any clinicopathological parameters. However, score values for MMP-1, -7 and -13, were significantly higher in AR-positive tumors than in AR-negative tumors. Likewise, when we considered the cellular type expressing each factor, we found that AR-positive tumors had a higher percentage of cases positive for MMP-1, -7, -11, and TIMP-2 in their malignant cells, as well as for MMP-1 in intratumoral fibroblasts. On the other hand, multivariate analysis demonstrated that patients with AR-positive tumors have a significant longer overall survival than those with AR-negative breast carcinomas (*p *= 0.03).

**Conclusion:**

Our results confirm that AR are commonly expressed in breast cancer, and are correlated with the expression of some MMPs and TIMP-2. Although we found a specific value of AR expression to be a prognostic indicator in breast cancer, the functional role of AR in these neoplasms is still unclear and further data are needed in order to clarify their biological signification in breast cancer.

## Background

In the last two decades, the molecular mechanisms related to the hormone dependence of breast tumors have been extensively investigated and the role of the estrogen and progesterone receptors (ER and PgR) in promoting breast cancer has been well documented. However, the role of androgens and their receptors (AR) in breast cancer etiology and progression has been less profoundly studied and remains an unanswered question [1,2]. There is evidence showing that androgens can directly stimulate the growth of human breast cancer cell lines [3]. In addition, both retrospective and prospective studies have reported statistically significant associations between increased levels of testosterone and higher breast cancer risk in both pre- and postmenopausal women [4-6]. Likewise, AR is expressed in approximately 70% to 90% of invasive breast cancers, a frequency comparable with or higher than the one reported for ER (70–80%) and PgR (50–70%) [1,7-10]. Although a relationship between AR and both ER and PgR status has been demonstrated [10-14], a significant percentage of tumors are positive for AR and negative for ER and PgR [13]. This finding reveals the independent expression of AR in human breast cancer. However, there are apparently divergent data on the biological and clinical signification of AR in breast cancer. AR have also been detected in a significantly higher percentage of AR-positive ductal carcinomas "in situ" (DCIS) adjacent to invasive carcinomas of the breast than in pure DCIS lesions [15], suggesting that AR correlates with tumor invasiveness, at least in the early phases of tumor progression. In invasive breast carcinomas, AR-positive tumors have been associated with a low or intermediate histological grade (G1, G2) [10,13,14,16,17]. In addition, certain types of breast carcinoma, even high grade ones, are typically ER- and PR-negative, but AR-positive; a typical example of such tumors is the apocrine breast carcinoma [18,19]. However, the expression level of both the AR gene and the AR protein in breast cancer was found to be positively correlated with axillary lymph node involvement [20]. In addition, it is remarkable that among the steroid hormone receptors, the androgen receptor is the best preserved one during metastases development and is expressed in the majority of metastatic tumors [8,21]. There is evidence as well indicating that AR/steroids are able to up-regulate matrix metalloproteases (MMPs), contributing to invasiveness via destruction of basement membrane and extracellular matrix [22,23]. Nevertheless, only a few studies have examined the impact of AR expression on patient prognosis in early breast cancer. Patients with AR-positive tumors were shown to have a significant trend toward longer relapse-free and/or overall survival in the univariate analysis than those patients with AR-negative tumors [1,16,24], but none in the multivariate analysis [1,16]. In addition, other studies have not found any significance of AR expression in predicting prognosis in breast cancer [20,25].

Since AR are expressed by an important percentage of breast carcinomas and there are evidences pointing their role in tumor progression, in the present study we analyze the tumor expression of AR, its relationship with clinicopathological characteristics, with several MMPs and their inhibitors (TIMPs) and with prognosis, in patients with breast cancer.

## Methods

### Patient characteristics and tissue specimen handling

This study comprised 111 women with a histologically confirmed diagnosis of breast cancer and treated between 1990 and 2001. We selected women with the following inclusion criteria: invasive ductal carcinoma, at least ten histopathologically-assessed axillary lymph nodes, and a minimum of five years of follow-up in those women without tumor recurrence. The exclusion criteria were the following: metastatic disease at presentation, prior history of any type of malignant tumor, bilateral breast cancer at presentation, having received any type of neoadjuvant therapy, development of loco-regional recurrence during the follow-up period, development of a second primary cancer, and absence of sufficient tissue in the paraffin blocks used for manufacturing the TMAs. From a total of 1053 patients fulfilling these criteria, we selected randomly a sample size of 111 patients, in accordance to four different groups of similar size and stratified with regard to nodal status and the development of metastatic disease, which were the key measure variables of this study. Thus, we included an important number of events in both node-negative and node-positive patients subgroups (half of the cases with distant metastasis during the follow-up period in each one of these subgroups) in order to warrant the statistical power of the survival analysis. Patients characteristics included in the two main groups, with or without distant metastases, are listed in Table 1. Histological grade was determined according to the criteria reported by Elston and Ellis [26].

**Table 1:** Relationship between AR expression and different clinicopathological parameters in 111 breast carcinomas.

**Patient and tumor characteristics**	N	Median (range)	p	N° positive cases (score >0) (%)	p
Total cases	111	69 (0–288)		83 (74.8)	

Age (years)			n.s.		n.s.

≤ 58	55	68(0–279)		44(80)	
>58	56	76(0–288)		39(69.6)	

Menopausal status			n.s.		n.s.

Premenopausal	29	92(0–279)		25(86.2)	
Postmenopausal	82	59(2–288)		58(70.4)	

Tumor size			n.s.		n.s.

T1	50	80(0–288)		40(80)	
T2	61	64(0–279)		43(70.5)	

Nodal status			n.s.		n.s.

Positive	52	53(0–273)		38(73.1)	
Negative	59	100(0–288)		45(76.3)	

Stage			n.s.		n.s.

I	32	71(0–273)		25 (78.1)	
II	51	64(0–243)		40 (78.4)	
III	28	83.5(0–288)		18 (64.3)	

Histologic grade			n.s.		n.s.

Well Dif.	30	66.5(0–273)		22(73.3)	
Mod. Dif.	57	88(0–288)		44(77.2)	
Poorly Dif.	24	40.5(0–267)		17(70.8)	

Estrogen receptor			n.s.		n.s.

Negative	52	55.5(0–273)		34(65.4)	
Positive	59	80(0–288)		49(83.1)	

Progesterone receptor			n.s.		n.s.

Negative	59	58(0–279)		41(69.5)	
Positive	52	76(0–288)		42(80.8)	

Desmoplastic reaction			n.s.		n.s.

No	35	48(0–270)		23(65.7)	
Yes	76	80.5(0–288)		60(78.9)	

Peritumoral inflammation			n.s.		n.s.

No	65	69.5(0–279)		48(75)	
Yes	46	86(0–288)		34(75.6)	

Tumor advancing edge			n.s.		n.s.

Expansive	49	50.5(0–270)		33(68.8)	
Infiltrating	62	84.5(0–288)		48(80)	

Vascular invasion			n.s.		n.s.

No	72	64.5(0–288)		52(72.2)	
Yes	39	81(0–279)		31(79.5)	

Women were treated according to the guidelines used in our institution. The study adhered to national regulations and was approved by our institution's Ethics and Investigation Committee. The end-point was death from tumor progression. The median follow-up period in patients without metastases was of 87.5 months, and of 52.7 months in patients with them.

### Tissue microarrays and immunohistochemistry

Routinely fixed (overnight in 10% buffered formalin), paraffin-embedded tumor samples stored in our pathology laboratory files were used in this study. Histopathologically representative tumor areas were defined on haematoxylin and eosin-stained sections and marked on the slides. Tumor tissue array blocks were obtained by punching a tissue cylinder (core) with a diameter of 1.5 mm through a histologically representative area of each 'donor' tumor block, which was then inserted into an empty 'recipient' tissue array paraffin block using a manual tissue arrayer (Beecker Instruments, Sun Praerie, Winconsin, USA) as described elsewhere [27]. Collection of tissue cores was carried out under highly controlled conditions. Areas of non-necrotic cancerous tissue were selected for arraying by two experienced pathologists (L.O. González and A. M. Merino). Two cores were employed for each case. From the 111 tumor samples available, three tissue array blocks were prepared, each one containing 37 tumors samples, as well as internal controls including four normal breast tissue samples from two healthy women who had undergone reductive mammary surgery.

Three composite high-density TMA blocks were designed, and serial 5-μm sections were consecutively cut with a microtome (Leica Microsystems GmbH, Wetzlar, Germany) and transferred to adhesive-coated slides. One section from each tissue array block was stained with H&E, and these slides were then reviewed to confirm that the sample was representative of the original tumor. Immunohistochemistry was done on these sections of TMA fixed in 10% buffered formalin and embedded in paraffin using a TechMate TM50 autostainer (Dako, Glostrup, Denmark). Antibodies for MMPs and TIMPs were obtained from Neomarker (Lab Vision Corporation, Fremont, CA, USA). The dilution for each antibody was established based on negative and positive controls (1/50 for MMP-2, -7, 14, and TIMP-2; 1/100 for 9, 13, TIMP-1 and -3; 1/200 for MMP-1, MMP-11); and anti-AR clone AR 441 (Dako) at a dilution of 1/50. The positive control was prostate carcinoma, previously tested. The negative control was DakoCytomation mouse serum diluted to the same mouse IgG concentration as the primary antibody.

Tissue sections were deparaffinized in xylene, and then rehydrated in graded concentrations of ethyl alcohol (100%, 96%, 80%, 70%, then water). To enhance antigen retrieval only for some antibodies, TMA sections were microwave-treated (H2800 Microwave Processor, EBSciences, East Granby, Connecticut, USA) in citrate buffer pH 6 (Target Retrieval Solution, Dako) at 99°C for 15 min. Endogenous peroxidase activity was blocked by incubating the slides in peroxidase-blocking solution (Dako) for 5 min. The EnVision Detection Kit (Dako) was used as the staining detection system. Sections were counterstained with hematoxilin, dehydrated with ethanol, and permanently coverslipped.

### TMA analysis

For each antibody preparation studied, the location of immunoreactivity, percentage of stained cells and intensity were determined. All the cases were semiquantified for each protein-stained area. An image analysis system made up by the Olympus BX51 microscope and soft analysis (analySIS^®^, Soft imaging system, Münster, Germany) was employed as follows: tumor sections were stained with antibodies according to the method explained above and counterstained with hematoxilin. There were different optical thresholds for both stains. Each core was scanned with a 400× power objective in two fields per core. Fields were selected searching for the protein-stained areas. The computer program selects and traces a line around antibody-stained areas (higher optical threshold: red spots), with the remaining, non-stained areas (hematoxilin-stained tissue with lower optical threshold) standing out as a blue background. Any field has an area ratio of stained (red) versus non-stained areas (blue). A final area ratio was obtained after averaging two fields. To evaluate immunostaining intensity we used a numeric score ranging from 0 to 3, reflecting the intensity as follows: 0, no staining; 1, weak staining; 2, moderate staining; and 3, intense staining. Using an Excel spreadsheet, the mean score was obtained by multiplying the intensity score (I) by the percentage of stained cells (PC) and the results were added together (total score: I × PC). This overall score was then averaged with the number of cores that were done for each patient. If there was no tumor in a particular core, then no score was given. In addition, for each tumor, the mean score of two core biopsies was calculated.

Furthermore, whole-tissue sections from tumor blocks from a subset of ten cases were compared with the corresponding TMA discs, regarding AR expression. These cases were selected randomly, and the obtained clinicopathological data were very similar to those from the whole series. Each whole-tissue section was scanned with a 400× power lens in ten different fields. Fields were selected searching for the protein-stained areas, such as it was described above. Previously, we described a similar validation study for the evaluated MMPs and TIMPs, in invasive breast cancer [28].

### Data analysis and statistical methods

Differences in percentages were calculated with the chi-square test. Immunostaining score values for each protein were expressed as median (range). Comparison of immunostaining values between groups was made with the Mann-Whitney or Kruskall-Wallis tests. For metastasis-free survival analysis we used the Cox's univariate method. Cox's regression model was used to examine interactions of different prognostic factors in a multivariate analysis. The SPSS 11.5 program was used for all calculations.

## Results

More than 2,800 determinations were performed on TMAs of cancer specimens from 111 patients with primary invasive ductal carcinoma of the breast and controls. Minimal internal variance of score data between duplicate tissue cores from the same patients was detected in the tissue arrays, showing a high agreement for each protein (*r *> 0.95 and *p *< 0.0001, for each protein). In the validation study there was a total concordance in the global expression, as well as in the intensity of immunostaining for AR, between TMA cases and the corresponding whole-tissue sections. In addition, there were highly significant correlations in the immunostaining scores between these two paired sets (*r *> 0.90 and *p *< 0.0001, for each protein).

**Figure 1:**
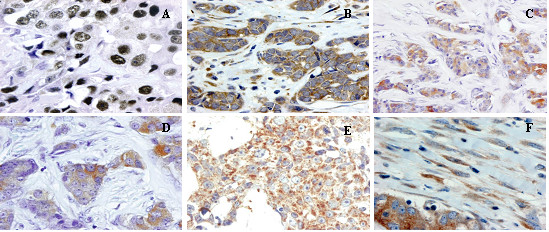
**Microphotographs representative of malignant cells positive for AR (A), MMP-1 (B), MMP-7 (C), MMP-13 (D) and TIMP-2 (E) and fibroblasts positive for MMP-1 (F).** Magnification 400×.

**Figure 2:**
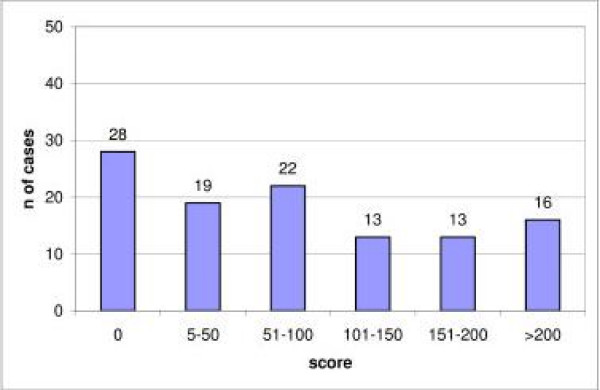
Distribution of AR score values in 111 breast carcinomas.

Figure 1 shows examples of tissue immunostained for the proteins evaluated. As it was expected, AR immunostaining was of nuclear localization. A total of 83 cases (74.8%) showed a positive immunostaining for AR, although with a wide variation in the immunostaining score values (Figure 2). There were only five cases with a score value of less than ten points (three with a score of 5, and two with a score of 8). Immunostaining for MMPs and TIMPs was localized predominantly in the cytoplasm of the malignant cells, but also in stromal cells in a considerable percentage of cases.

There were not any significant associations between the total immunostaining scores for AR and clinicopathological parameters such as tumor size, lymph node involvement, stage, histological grade, high Nottingham prognostic index, infiltrating edge, vascular invasion, desmoplastic reaction or peritumoral inflammation, ER or PgR status (Table 1).

In the present study we also investigated the possible relationship between AR expression and both MMPs and TIMPs expression in tumors, which have been associated with an aggressive behaviour and a poor prognosis in breast cancer patients. Our data demonstrated some significant associations. Thus, score values for MMP-1, -7 and -13, were significantly higher in AR-positive tumors than in AR-negative tumors (Table 2). Likewise, when we considered the cellular type expressing each factor, we found that the scores for AR were significantly higher in malignant cells from tumors positive for MMP-1, -7, -11, and TIMP-2, as well as for MMP-1 in intratumor fibroblasts (Table 3). However, there was not any significant association between MMPs or TIMPs expression by mononuclear inflammatory cells and the AR status of the tumors (Table 3).

**Table 2:** Relationship between AR and MMPs and TIMPs expression in 111 breast carcinomas.

	**AR score values (median (range))**		
**Factor**	**AR negative**	**AR positive**	**p**

MMP-1	117(0–285)	140(0–285)	0.01
MMP-2	0(0–136)	0(0–246)	n.s.
MMP-7	65.1(0–267.5)	142(0–270)	0.002
MMP-9	62.5(0–176)	77(0–273)	n.s.
MMP-11	150(0–279)	157.6(0–277.7)	n.s.
MMP-13	52.8(0–234)	63(0–192.3)	0.04
MMP-14	83.8(0–254)	83(0–261)	n.s.
TIMP-1	135(0–276)	140(0–285)	n.s.
TIMP-2	104(0–243)	83(0–243)	n.s.
TIMP-3	65.3(0–272.4)	116(0–271.3)	n.s.

**Table 3:** Relationship between the expression of AR, MMPs and TIMPs by each cellular type in breast cancer.

**Factor**	**N**	**AR score values median (range)**	**p value**
**MMP-1**			

TC (-) *vs*. (+)	7/104	0(0–70)/80(0–288)	0.004
FC (-) *vs*. (+)	14/97	2.5(0–225)/80(0–288)	0.03
MIC (-) *vs*. (+)	30/81	53(0–225)/81(0–288)	n.s.

**MMP-2**			

TC (-) *vs*. (+)	70/41	59.5(0–279)/90(0–288)	n.s.
FC (-) *vs*. (+)	83/28	69(0–288)/88(0–270)	n.s.
MIC (-) *vs*. (+)	108/3	68.5(0–288)/66(0–132)	n.s.

**MMP-7**			

TC (-) *vs*. (+)	8/103	0(0–156)/80(0–288)	0.01
FC (-) *vs*. (+)	27/84	68(0–267)/74.5(0–288)	n.s.
MIC (-) *vs*. (+)	50/61	66(0–267)/81(0–288)	n.s.

**MMP-9**			

TC (-) *vs*. (+)	27/84	48(0–270)/80.5(0–288)	n.s.
FC (-) *vs*. (+)	93/18	68(0–288)/83.5(0–270)	n.s.
MIC (-) *vs*. (+)	98/13	80(0–279)/30(0–288)	n.s.

**MMP-11**			

TC (-) *vs*. (+)	11/100	8(0–168)/80(0–288)	0.03
FC (-) *vs*. (+)	33/78	53(0–264)/81(0–288)	n.s.
MIC (-) *vs*. (+)	72/39	68(0–270)/87(0–288)	n.s.

**MMP-13**			

TC (-) *vs*. (+)	29/82	48(0–225)/74.5(0–288)	n.s.
FC (-) *vs*. (+)	54/57	56(0–267)/81(0–288)	n.s.
MIC (-) *vs*. (+)	70/41	64.5(0–279)/92(0–288)	n.s.

**MMP-14**			

TC (-) *vs*. (+)	10/101	64.5(0–273)/69(0–288)	n.s.
FC (-) *vs*. (+)	21/90	92(0–273)/66.5(0–288)	n.s.
MIC (-) *vs*. (+)	50/61	64.5(0–273)/81(0–288)	n.s.

**TIMP-1**			

TC (-) *vs*. (+)	6/105	0(0–225)/72(0–288)	n.s.
FC (-) *vs*. (+)	56/55	70(0–288)/69(0–273)	n.s.
MIC (-) *vs*. (+)	81/30	64(0–279)/96(0–288)	n.s.

**TIMP-2**			

TC (-) *vs*. (+)	13/98	0(0–174)/80(0–288)	0.01
FC (-) *vs*. (+)	59/52	80(0–270)/63(0–288)	n.s.
MIC (-) *vs*. (+)	63/48	80(0–288)/68.5(0–279)	n.s.

**TIMP-3**			

TC (-) *vs*. (+)	15/96	48(0–210)/76(0–288)	n.s.
FC (-) *vs*. (+)	42/69	50.5(0–225)/88(0–288)	n.s.
MIC (-) *vs*. (+)	53/58	64(0–279)/87(0–288)	n.s.

**Figure 3:**
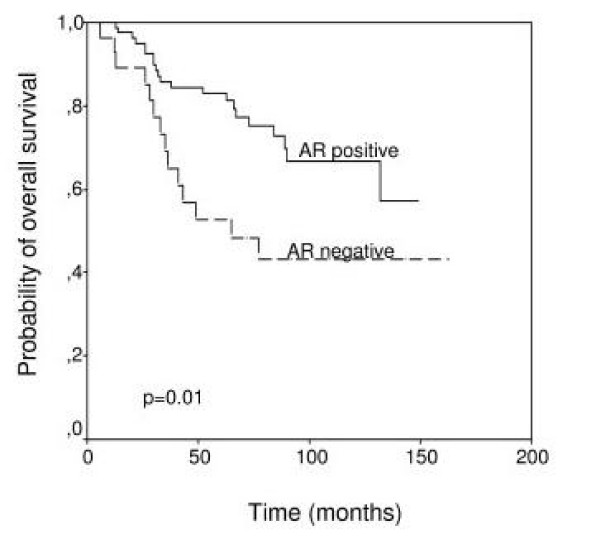
Relapse-free survival and overall survival as a function of AR values in 111 breast carcinomas.

We initially investigated the possible association between each immunostaining score value for AR, as cut-off points, and relapse-free survival. We found that none of these cut-off points were significantly associated with relapse-free survival in our patient population (data not shown). However, our results demonstrated that when patients were dichotomized in two different groups with regard to the more optimal cut-off point of score values for AR (score = 0 *v.s*. score > 0), patients with AR-positive tumors had a significantly longer survival than patients with AR-negative tumors (*p *= 0.01) (Figure 3), but there was no difference regarding the occurrence of distant metastases (data no shown). In addition, and in accordance with previous studies indicating the prognostic value of AR expression in ER-negative tumors, we investigated that value in the subset of 59 ER-negative tumors included in the present study, but we could not find any significant value of AR expression able to predict either relapse-free or overall survival in the corresponding patients (data not shown). Multivariate analysis according to Cox model demonstrated that tumor stage (stage II: relative risk (RR) (confidence interval): 3.46(1.11–10.78); stage III: 7.29(2.37–22.41); *p *< 0.001) and PgR status (positive: 0.19(0.08–0.49), *p *< 0.001) were significantly associated with overall survival. Multivariate analysis also confirmed that patients with AR-positive carcinomas had a significant longer overall survival than those with AR-negative breast neoplasms (AR-positive: 0.46(0.23–0.93), *p *= 0.03).

## Discussion

Our results confirmed previous biochemical and immunohistochemical studies indicating that AR are expressed in a considerable proportion of breast carcinomas [1,7-10]. We also found that AR expression in breast carcinomas is highly variable because of tumor heterogeneity. We determined that there are some correlations between AR and MMPs expression. However, AR expression was not related with the occurrence of distant metastases, but instead associated with a longer overall survival in breast cancer patients.

Contrary to other studies, we found no significant differences between AR expression and the clinicopathological characteristics of the tumors, such as histological grade [10,13,14,16,17,29], axillary lymph node involvement [20], ER or PgR status [10-14]. Nevertheless, we found a positive and significant relationship between AR expression and the expression of parameters potentially indicative of invasiveness, such as MMP-1, -7 and -13. This association could be relevant because it is now known that MMPs promote metastases not only by modulating the remodelling of extracellular matrix, but because MMPs are able to impact *in vivo *on tumor cell behaviour as a consequence of their ability to cleave growth factors, cell surface receptors, cell adhesion molecules, and chemokines/cytoquines [30-33]. Furthermore, by cleaving pro-apoptotic factors, MMPs are able to produce a more aggressive phenotype via generation of apoptotic resistant cells [34]. MMPs may also regulate cancer-related angiogenesis, both positively through their ability to mobilize or activate pro-angiogenic factors [35], and negatively via generation of angiogenesis inhibitors, such as angiostatin and endostatin, cleaved from large protein precursors [36]. In addition, it is now understood that TIMPs are multifactorial proteins also involved in the induction of proliferation and the inhibition of apoptosis [37,38]. In a prior report we found an increase in AR expression in the transition from pure DCIS to DCIS adjacent to the invasive component of breast carcinoma, which led us to consider that there could be a relationship between both AR and MMPs/TIMPs expression in breast carcinoma [39]. There are evidences indicating the existence of a steroid regulation of the gelatinases (MMP -2 and MMP -9) in both breast [40,41] and prostate cancer [42,43]. Likewise, it is of note that the expression of MMP -13 (collagenase-3), which has been associated with the microinvasive component of "in situ" carcinomas [44], has been found to be up-regulated by androgens in prostate cancer derived the cell line LNCaP [23]. Nevertheless, the regulation of MMPs production by androgens seems to be a quite complex process. Thus, experimental studies showed that androgens, via AR-Ets, negatively regulate the expression of interstitial collagenase (MMP -1), stromelisin-1 (MMP -3), and matrilysin -1 (MMP -7) [45]. Even so, in despite of assuming an association between AR expression and some MMPs/TIMPs production in the context of breast cancer, we could not determine any significant relationship between AR status and the occurrence of distant metastases. This may be due to the consideration of the cellular type expressing each factor in the tumor scene. In accordance with other authors, we found that AR immunoreactivity is localized in the nuclei of tumor cells and no stromal staining was observed [1,13]. However, there is a biological variability with regard to the cellular type expressing MMPs or TIMPs (cancerous cells and/or stromal cells -fibroblasts or mononuclear inflammatory cells-). When we considered this morphological aspect, we found that AR-positive tumors had a higher percentage of cases positive for MMP-1, -7, -11, and TIMP-2 in their malignant cells, when compared to AR-negative tumors. The only association with AR-positive status in stromal cells was for MMP-1 in intratumor fibroblasts. We believe that these findings could explain our results pointing the lack of any significant association between AR status and the occurrence of distant metastases because, such as it was recently reported from our group, the expression of these MMPs and the TIMP-2 correlate with distant metastases mainly when those are expressed by stromal cells [15,28]. Thus, our results led us to consider the existence of a regulation of MMPs/TIMPs expression via AR in the same tumor cells, but without a significant influence in the development of distant metastases. We considered that both AR and MMPs/TIMPs expression could be more important in the early phases of tumor progression, but less in primary invasive breast carcinomas.

On the other hand, our data demonstrated that the AR status correlates significantly and independently with overall patient survival. Other authors have also found that breast cancer patients with AR-negative tumors show a trend toward a shorter overall survival than those patients with AR-positive tumors [24]. It has been proposed that this trend may be secondary to the AR-positive tumors' capability to retain a hormone-sensibility that confers a low biological aggressiveness. In fact, among the steroid hormone receptors, AR is the best preserved one during metastases development and is expressed in the majority of metastatic tumors [21]. Furthermore, the effects of tamoxifen [46] and medroxyprogesterone acetate are mediated by AR [47]. A recent study showed that reduced levels of AR or impaired AR function contribute to the failure of medroxyprogesterone acetate therapy, potentially due to the abrogation of the inhibitory effect of AR on ER signaling [48]. In addition, Aggof et al. have reported a significant association in the univariate analysis (p = 0.049) between AR expression and relapse-free survival in patients with ER-negative tumors (n = 57), but none with overall survival [16]. In the present study we did not find this association. Nevertheless, there are possible explanations for that discrepancy with our results due to differences in the studied patient populations. Thus, although we have a similar number of patients with ER-negative tumors (n = 59), it is of note that our study included a higher number of events (tumor relapses) (61%) than in the study of Aggof et al, (33%), because our population was selected stratifying on the basis of the occurrence of distant metastases. Likewise, in our study we applied different criteria for patient selection, such as ductal being the chosen histological type, considering distant metastases as the only type of tumor recurrence, and including only T1 and T2 tumors. On the other hand it is remarkable that Schippinger et al., did not find in their multivariate analysis any independent prognostic value for AR-expression in patients with metastatic breast cancer [25]. Nevertheless, this patient population differs clinically of that of non-metastatic breast cancer included in our study. Even so, the latter finding seems to indicate that the prognostic significance of AR status may be lost once distant metastatic disease occurs.

## Conclusion

Our results confirm that AR are commonly expressed in breast cancer, and correlate with the expression of some MMPs and TIMP-2. Although we found a value of AR expression to be a prognostic indicator in breast cancer, the functional role of AR in these neoplasms is still unclear and further data are needed to determine their biological signification in breast cancer, i.e. if AR could be used as a marker for efficiency of endocrine therapy and for new hormonal therapeutic strategies in women with ER-negative carcinomas, as well as on the prognostic signification of AR microsatellites polymorphism in breast cancer, which could affect the transactivation capacity of the receptor [49], and whether or not patients with ER-negative, PR-negative, but AR-positive cancers behave differently from those with triple negative breast carcinomas.

## Competing interests

The authors declare that they have no competing interests.

## Authors' contributions

LOG carried out the immunohistochemical analysis. MDC carried out the immunohistochemical assays and participated in drafting the manuscript. JV participated in the study's design and performed the statistical analysis. SJ carried out the immunohistochemical analysis. RS participated in the recording of clinical data. ACA participated in the recording of clinical data and in the immunohistochemical analysis. JCR conceived the study, and participated in its design and coordination and helped drafting the manuscript. MLL participated in the recording of clinical data. FJV conceived the study, participated in its design and coordination, helped drafting the manuscript and revising it critically for important intellectual content.

All the authors read and approved the final manuscript.

## Pre-publication history

The pre-publication history for this paper can be accessed here:


